# Molecular Testing of Atypical Thyroid Nodules with Corresponding Surgical Correlation: Five-Year Retrospective Review in Veterans Population

**DOI:** 10.7759/cureus.22536

**Published:** 2022-02-23

**Authors:** Diane M Carr, Stephen Mastorides, Corinne Stobaugh, George Carlton, Lauren DeLand, Andrew Borkowski

**Affiliations:** 1 Pathology and Laboratory Medicine, University of South Florida, Tampa, USA; 2 Pathology and Laboratory Medicine, James A. Haley Veterans' Hospital, Tampa, USA; 3 Nursing, James A. Haley Veterans' Hospital, Tampa, USA

**Keywords:** indeterminate, study, retrospective, correlation, molecular, thyroid, cytology

## Abstract

Objective

We report the results of a retrospective five-year study within a veteran population aimed at correlating abnormal thyroid fine-needle aspiration (FNA) diagnosis with associated molecular testing to the histology of the surgical resection.

Methods

A retrospective analysis of abnormal thyroid FNAs with associated molecular testing and surgical outcome was conducted from January 1, 2015 to December 31, 2020. Aspirates were classified using the Bethesda system for reporting thyroid cytopathology, including atypia of undetermined significance/follicular lesion of undetermined significance (AUS/FLUS), follicular neoplasm/suspicious for follicular neoplasm (FN/SFN), suspicious for malignancy (SM), and malignant. Pertinent data, including patient demographics, imaging, and ancillary testing were reviewed. A thyroid cancer mutation panel assessing the most common mutations and rearrangements associated with neoplasia was utilized. The results of molecular testing were directly compared and correlated with final cytological and histological diagnosis.

Results

A total of 1850 thyroid aspirates were performed, 200 of which were given an abnormal cytologic diagnosis. Thirty-six samples were submitted for molecular testing and subsequent surgical follow-up. Four were called malignant on cytology. 32 were placed in an indeterminate category (89%). Within indeterminate cases: 53% exhibited positive molecular mutations (n=17), 34% no mutation detected (n=11), and 13% insufficient quantity for testing (n=4). Upon surgical resection in the mutation-positive group: 18% had no malignancy (n=3), and the remaining 82% were positive for malignancy (n=14). Mutations in the histologically malignant group included: 57% BRAF (n=8), 21% NRAS (n=3), 7% HRAS (n=1), 7% KRAS (n=1), and 7% PAX8/PPAR gamma (n=1). In indeterminate cases with no mutation detected, 10 cases were found to be benign, and one case of malignancy was diagnosed. The probability of indeterminate diagnosis in combination with no mutation yielded a 91% chance of benign entity and 9% chance of malignancy. We demonstrated 93% sensitivity and 91% negative predictive value (NPV) for the risk of malignancy in indeterminate cytology specimens with ancillary molecular testing. There was 77% specificity and 82% positive predictive value (PPV) for our data set.

Conclusions

In indeterminate samples, the detection of a mutation was highly predictive of malignancy and a strong indicating factor for surgery with a high sensitivity and NPV. Molecular testing refined or established the diagnosis in 89% of the cases. Our results indicate that molecular testing of thyroid nodules enhances the accuracy of FNA cytology and the subsequent surgical outcome.

## Introduction

Thyroid nodules are among the most common diseases of the endocrine system, with an estimated prevalence of palpable nodules in the United States of 4%-7% and a prevalence of incidental ultrasonography detectable nodules between 19%-67%. These numbers increase annually worldwide. Most thyroid nodules can be managed conservatively due to their benign nature, but approximately 5%-15% of nodules examined by ultrasound and fine-needle aspiration (FNA) cytology lead to a malignant diagnosis [1-3]. Distinguishing between benign and malignant nodules to ensure that the patient receives appropriate treatment can challenge both a clinician and pathologist. One of the most reliable, safe, and commonly used diagnostic tests for thyroid nodules is FNA, a procedure in which cells from the nodule are collected using a high gauge thin needle, followed by staining and cytological examination of the cells with microscopic evaluation [4-6].

In most cases, FNA cytology can accurately diagnose a benign or malignant lesion; however, 10%-40% of all FNAs can lead to an indeterminate diagnosis. The ambiguous nature of these specimens can be a significant limitation to thyroid FNA, making the management of these indeterminate thyroid nodules challenging. Several factors, such as nuclear atypia, hypercellularity, and crowding, can lead to an indeterminate diagnosis. The Bethesda System for Reporting Thyroid Cytology (TBSRTC) standardizes the reporting systems used by cytopathologists. Indeterminate thyroid lesions are usually incorporated into the following three groups: atypia of undetermined significance/follicular lesion of undetermined significance (AUS/FLUS, Bethesda category III); follicular neoplasm/suspicious for follicular neoplasm (FN/SFN, Bethesda category IV); and suspicious for malignancy (SM, Bethesda category V) [7]. Many of the AUS/FLUS and FN/SFN aspirates are ultimately benign and are classified with a diagnosis of follicular adenoma or nodular hyperplasia on surgical resection [8]. In these cases, extensive surgery is unnecessary as a more conservative approach is often better for the patient. Ancillary molecular testing on fine-needle aspirates can be a valuable tool to determine whether clinical follow-up or surgery is warranted for these patients. Papillary carcinoma, the primary malignancy found in most abnormal thyroid cases, usually carries a BRAF, RET/PTC, or RAS mutation. These mutations are found in more than 70% of papillary carcinomas [9-10]. Follicular carcinoma, the second most common type of thyroid cancer, exhibits either RAS or PAX8/PPAR gamma mutations. Many studies in recent years have shown that detecting these molecular somatic mutations in thyroid FNA samples can improve the diagnostic accuracy of abnormal lesions. Herein, we report the results of a retrospective five-year review within the military veteran's population exhibiting that molecular testing adds significantly to the value of cytology and provides valuable information for both the pathologist and clinician regarding outcome.

## Materials and methods

A retrospective analysis of abnormal and indeterminate thyroid FNAs with associated molecular testing results and the final surgical outcome was conducted at the James A. Haley Veterans Administration facility located in Tampa, FL, over five years from January 1, 2015 to December 31, 2020.

An electronic search of the cytopathology database record was performed to identify abnormal thyroid aspirates with material submitted for molecular testing and subsequent surgical follow-up. All aspirates were classified using the Bethesda system for reporting thyroid cytopathology. The cases included AUS/FLUS, FN/SFN, SM, and positive for malignancy. The aspirates were examined by independent board-certified pathologists and often reviewed in consultation with an additional pathologist. The Computerized Patient Record System was employed to collect pertinent data, including patient demographics, imaging characteristics, and ancillary testing. Our institution utilized ancillary testing with a thyroid cancer mutation panel performed by Quest Diagnostics. The thyroid mutation panel assesses seven of the most common mutations or rearrangements associated with thyroid neoplasia. The BRAF codon 600 mutation and RET/PTC1 and RET/PTC3 rearrangements are highly associated with papillary thyroid cancer, the PAX8-PPAR gamma translocation with follicular carcinomas and mutation in HRAS, KRAS or NRAS most often occur in follicular neoplasms [4-6]. The BRAF assay tests for codon 600 BRAF mutation using a DNA-based mutation-specific polymerase chain reaction (PCR) method, with an approximate sensitivity of 0.1% mutation-bearing cells in a mixed sample. The RAS panel tests for mutations in codons 12 and 13 (exon 2) and codon 61 (exons 3) of HRAS, KRAS and NRAS by a DNA-based sequencing method, with an approximate sensitivity of 3% to 6% mutation-bearing cells in a mixed sample. This assay will not detect the rare RAS mutations in codons besides 12, 13 and 61. RET/PTC1 and RET/PTC3 rearrangements are detected using a reverse transcription PCR assay. The lower limit of sensitivity is approximately 2.5% mutation-bearing cells in a mixed sample but can vary due to RNA preservation. PPARG transcripts in relation to a control gene are detected by an RNA-based reverse transcription PCR-based method, with elevated PPARG levels indicative of PAX8/PPARG translocation.

Practice guidelines from the American Thyroid Association recommend that physicians consider molecular markers as aids in clinical management and follow-up [8-10]. The results of the molecular diagnostic testing were directly compared and correlated with the final cytological and histological diagnosis. The purpose of this study was to evaluate the utility of molecular testing results used in conjunction with cytopathologic fine-needle aspiration diagnosis and correlate with the subsequent surgical resection outcome. Based on the surgical resection diagnosis, we calculated the sensitivity, specificity, positive predictive value (PPV), and negative predictive value (NPV) for malignancy of indeterminate cytology cases (AUS/FLUS, FN/SFN, SM) in conjunction with molecular assay results.

## Results

**Figure 1:**
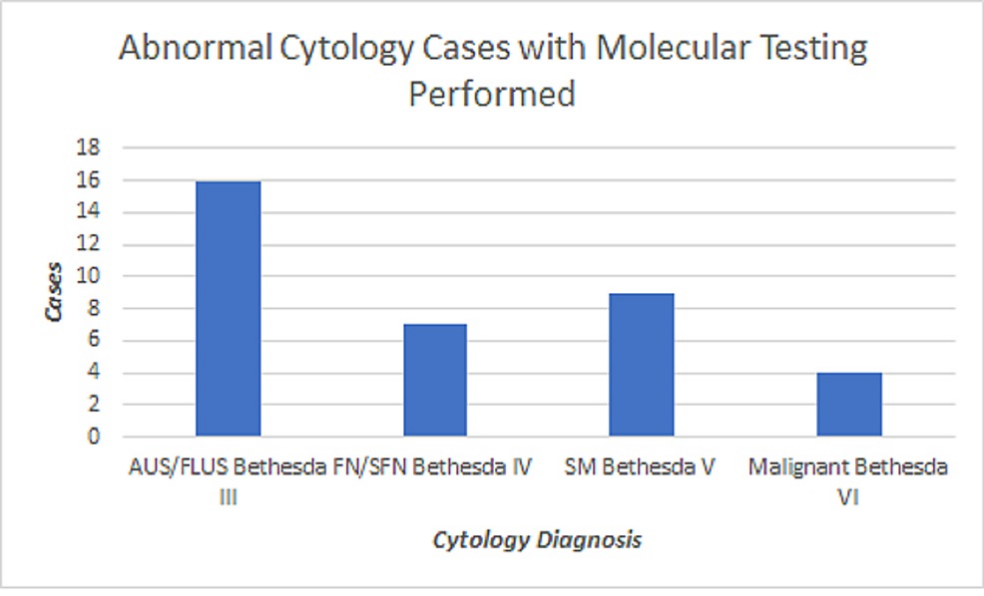
Fine-needle aspiration cytology reporting of the 36 thyroid cases, according to the Bethesda system with molecular testing performed. AUS/FLUS: atypia of undetermined significance/follicular lesion of undetermined significance; FN/SFN: follicular neoplasm/suspicious for follicular neoplasm; SM: suspicious for malignancy.

A total of 1850 thyroid aspirates were performed at the James A. Haley Veterans Administration facility from January 1, 2015, through December 31, 2020. Of those aspirates, a total of 200 cases were designated with an abnormal cytologic diagnosis. Within this subgroup of abnormal cytological cases, 36 patient samples were submitted for molecular testing and had a subsequent surgical follow-up (Figure 1). The samples were obtained from 30 males and 6 females, ranging in age from 28 to 78 years old. The preoperative diagnosis were AUS/FLUS (n=16), FN/SFN (n=7), SFM (n=9), and Malignant (n=4). Of the 36 specimens sent for thyroid cancer mutation panel, 12 had no mutation detected in the FNA material, four came back as quantity not sufficient to perform testing, and the remaining 20 exhibited a molecular mutation revealing an overall suspicious result. Subsequently, 15 lobectomies and 21 total thyroidectomies were performed. The resection specimens showed nodular hyperplasia (n = 2), benign adenoma (n = 12), and non-invasive follicular thyroid neoplasm with papillary-like nuclear features (NIFTP) (n=1). Malignancies included conventional papillary thyroid carcinoma (PTC) (n= 13), micro-invasive PTC (n = 1), follicular variant of papillary thyroid carcinoma (FVPTC) (n = 4), oncocytic variant of PTC (n = 1), follicular carcinoma (FC) (n=1), and medullary carcinoma (MC) (n = 1).

**Figure 2:**
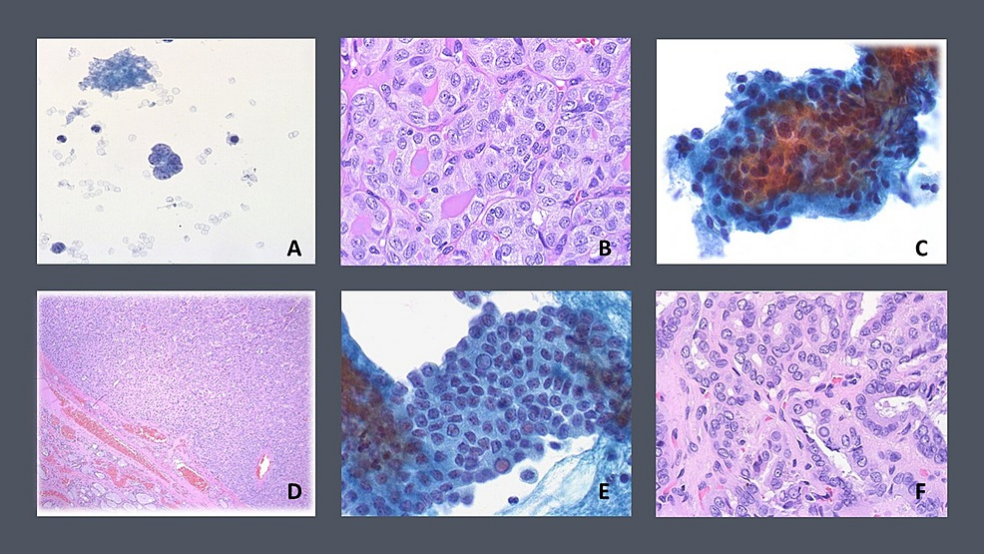
A) Example of AUS/FLUS indeterminate cytology which tested positive for NRAS mutation. B) Surgical resection ultimately revealed FVPTC on histologic evaluation C) Example of FN/SFN indeterminate cytology which tested negative for a molecular mutation. D) Surgical resection ultimately revealed benign oncocytic adenoma on histologic evaluation. E) Example of SM indeterminate cytology which tested positive for BRAF mutation. F) Surgical resection ultimately revealed conventional PTC on histologic evaluation. AUS/FLUS: atypia of undetermined significance/follicular lesion of undetermined significance; FVPTC: follicular variant of papillary thyroid carcinoma; FN/SFN: follicular neoplasm/suspicious for follicular neoplasm; SM: suspicious for malignancy; PTC: papillary thyroid carcinoma.

The presence of any mutation was a strong indicator of malignancy, with 17 out of 20 (85%) mutation-positive nodules found to be malignant after surgery. Whereas cytology alone only placed four specimens in the definitively malignant category previously, the combination of cytology and positive molecular testing showed a significant improvement in the accuracy of a definitive diagnosis of malignant thyroid nodules. BRAF was the most frequent mutation (n=11). Eight of the specimens exhibiting this mutation were subsequently revealed to be conventional PTC on histological evaluation (Figure 2, bottom panel); two of the specimens were diagnosed as FVPTC, and one as a benign entity. These included two cases that were positive for malignancy on original cytology and nine that fell within one of the indeterminate cytology categories (AUS/FLUS, FN/SFN, SM). RAS mutations were the second most common mutation type to include NRAS (n=4), HRAS (n=3), and KRAS (n = 1). After surgery, two of the NRAS positive nodules and the KRAS positive nodule yielded a conventional PTC diagnosis. One NRAS positive nodule was classified as a FVPTC (Figure 2). One HRAS positive nodule on surgical histology was deemed a follicular carcinoma, and another a medullary carcinoma.

**Figure 3:**
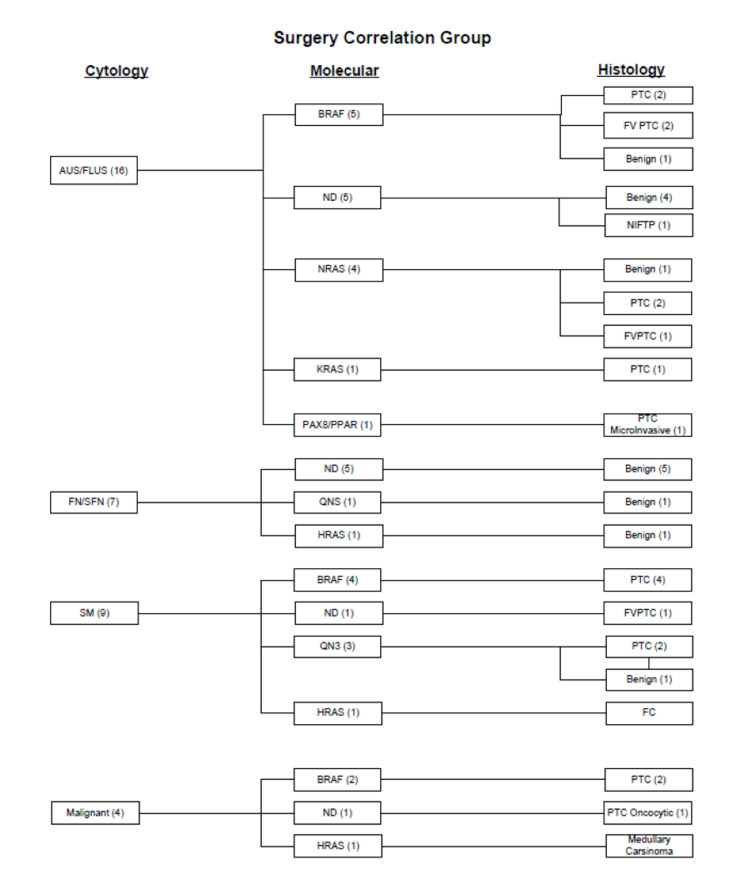
Correlation between cytology, molecular findings, and histological diagnosis in the surgery correlation group. AUS/FLUS: atypia of undetermined significance/follicular lesion of undetermined significance; FN/SFN: follicular neoplasm/suspicious for follicular neoplasm; SM: suspicious for malignancy; PTC: papillary thyroid carcinoma; FVPTC: follicular variant of papillary thyroid carcinoma; FC: follicular carcinoma; NIFTP: non-invasive follicular thyroid neoplasm with papillary-like nuclear features; ND: mutation not detected; QNS: quantity not sufficient.

Interestingly, two cases, one NRAS mutation positive and one HRAS positive, along with the previously mentioned BRAF positive case, were the only three where the presence of a mutation found in the abnormal cytology specimen did not correlate with a malignancy on surgical resection. Of these abnormal cytology specimens with a positive RAS mutation, the medullary carcinoma was the only previous specimen with a definitive positive for malignancy designation on cytology diagnosis alone; the others were placed in one of the indeterminate categories (AUS/FLUS, FN/SFN, or SM). The single nodule with PAX8/PPAR gamma rearrangement (n=1) was found to be a micro-invasive PTC. In the cases with a molecular determination of mutation not detected (ND) (n=12), one was a FVPTC, one an oncocytic variant of PTC, one a non-invasive follicular thyroid neoplasm with papillary like nuclear features NIFTP, and nine benign entities. On cytological diagnosis alone, one of the specimens without a detectable mutation was classified as positive for malignancy; the remaining 11 cases fell into one of the indeterminate categories (AUS/FLUS, FN/SFN, SM). In the category where molecular testing could not be completed due to insufficient quantity (n = 4), two surgical resection specimens were designated conventional PTC, and two were classified benign. All four were previously designated in a cytologically indeterminate category prior to molecular testing and surgical resection. Overall, the detection of a mutation had a strong correlation with a malignant outcome (Figure 3).

**Figure 4:**
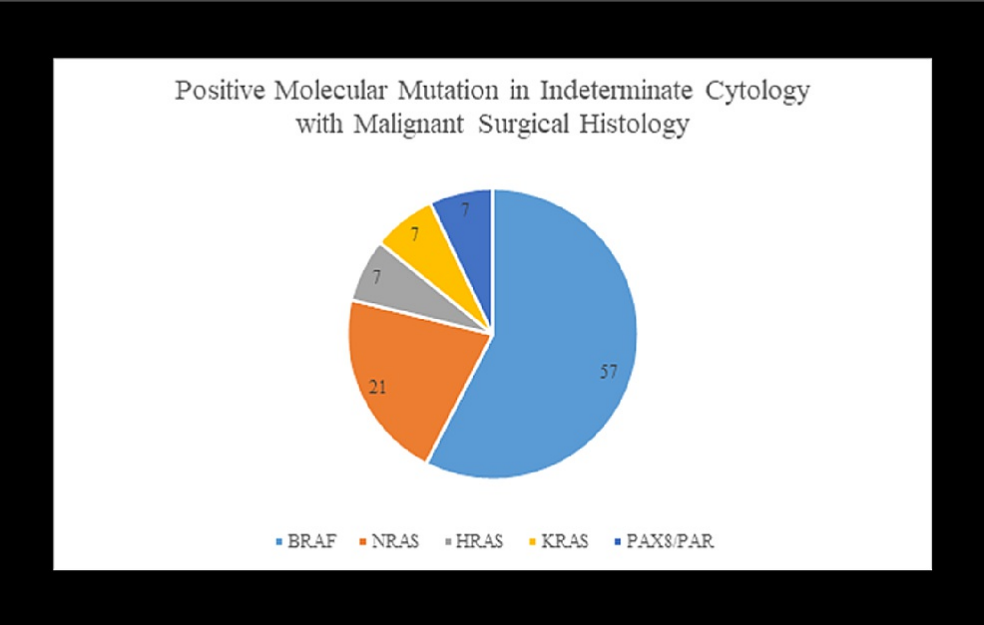
Percentage of mutations in positive surgical resections.

**Figure 5:**
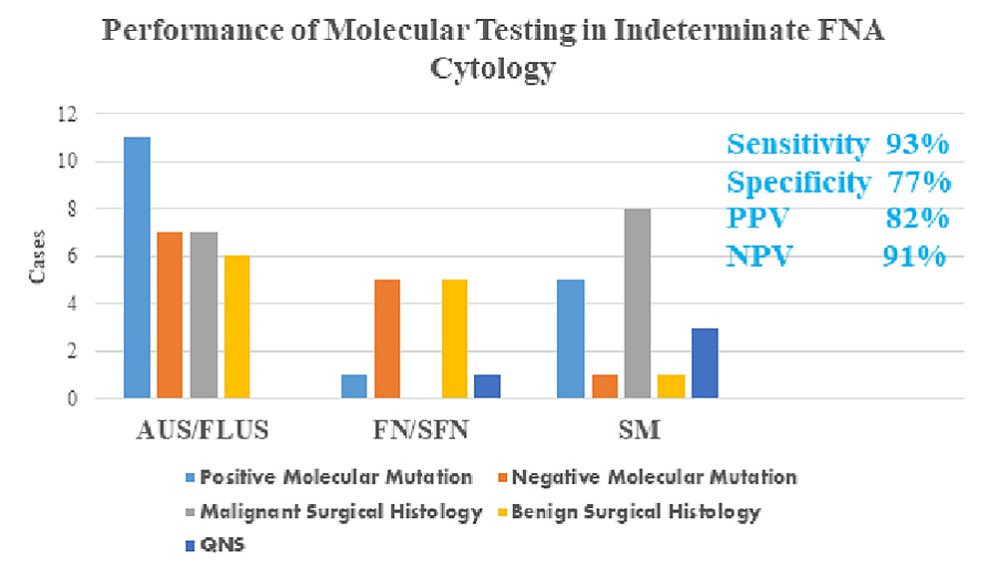
FNA category with molecular findings and surgical outcomes. FNA: fine-needle aspiration; AUS/FLUS: atypia of undetermined significance/follicular lesion of undetermined significance; FN/SFN: follicular neoplasm/suspicious for follicular neoplasm; SM: suspicious for malignancy; QNS: quantity not sufficient.

Molecular testing contributed to better predicting the probability of both malignant and benign nodules with indeterminate cytology diagnosis. Among the specimens with an initial fine needle aspiration performed, four of the 36 cases were definitively called positive for malignancy (11%). The remaining 89% were placed in an indeterminate cytology category (n=32). From these 32 cases, 53% exhibited positive molecular mutations (n=17), 34% had no mutation detected (n=11), and 13% could not have testing completed due to insufficient quantity (n=4). Of the indeterminate cytology cases with positive molecular mutation testing, 18% were found to have no malignancy upon surgical resection (n=3). The remaining 82% were found to be positive for malignancy upon surgical resection (n=14). In the cytologically indeterminate cases with subsequent positive surgical diagnosis, 57% exhibited BRAF mutation (n=8), 21% had NRAS mutation (n=3), 7% exhibited HRAS mutation (n=1), 7% had KRAS mutation (n=1), and 7% showed a PAX8/PPAR gamma mutation (n=1) (Figure 4). In the indeterminate cytology cases with no molecular mutation detected, i.e., negative molecular testing, 10 surgical resection cases were found to be benign, and one case of malignancy was found. The probability of an indeterminate cytology diagnosis in combination with no molecular mutation detected yielded a 91% chance of a benign entity and a 9% chance of malignancy due to this singular event in our study. We demonstrated a high sensitivity of 93% and negative predictive value (NPV) of 91% for the risk of malignancy in an indeterminate cytology specimen with ancillary molecular testing. Molecular testing achieved 77% specificity and 82% positive predictive value (PPV) when applied to samples of indeterminate cytology (Figure 5).

## Discussion

We report here the results of a five-year retrospective study in a veteran population aimed at correlating abnormal cytologic fine-needle aspiration diagnosis with associated molecular genetic testing to the histology of the subsequent surgical resection. Similar to Nikiforov et al., we show that the testing for a panel of molecular mutations significantly improves the accuracy of diagnosis, particularly for samples with indeterminate cytology [2]. In this category, the positive detection of a mutation was highly predictive of malignancy and a strong indicating factor for surgery with a high NPV and an intermediate PPV for malignancy. Many FNA patients can benefit from molecular testing, which can accurately predict malignancy and avoid the cost of repeat FNA procedures [2].

As expected, BRAF mutation was the most common and had a 91% positive predictive value for PTC. RAS mutations were the second most common finding, also displaying a high diagnostic value. This current review indicates that finding a RAS mutation in an FNA sample conferred a 75% probability of malignancy, including a 50% probability of PTC in our population. In addition, three cases exhibited a false positive molecular test with mutations found in benign follicular adenomas and some hyperplastic nodules. These may indicate precursor lesions, and surgical removal may be justified to prevent progression [10]. However, this result shows that molecular testing should not be used in isolation to guide clinical management, but that the preoperative detection of mutations can provide helpful prognostic information and additional targeted therapies even when cytology is not definitive. The final histopathologic diagnosis remains the gold standard for comparison with preoperative cytology. Molecular testing refined or established the diagnosis in 32 of the 36 cases in this review.

## Conclusions

In this five-year retrospective review, we examined the correlation of atypical and indeterminate thyroid cytology diagnosis with the molecular findings and subsequent surgical outcomes in our veteran population. We compared the indeterminate case diagnoses with the corresponding molecular mutation findings and the final surgical diagnoses. After examining the five years of data, our results and analysis indicate that molecular testing of thyroid nodules enhances the accuracy of FNA cytology and aids in the final surgical determination. Our results within the veterans' population are comparative with several similar studies performed recently by outside institutions that also emphasize the ability of molecular testing for mutations to both improve FNA diagnosis and access risk of malignancy in indeterminate thyroid cases.
